# A community-based pragmatic, controlled trial for preventing and reducing oral diseases among 1–6-year-old children visiting Anganwadi centers, under the Integrated Child Development Scheme, India

**DOI:** 10.1186/s12889-019-7874-y

**Published:** 2019-12-03

**Authors:** Ashima Goyal, Ashoo Grover, Krishan Gauba, Arpit Gupta, Nishant Mehta, Sourabh Dutta, R. M. Pandey, Ashish Joshi, J. S. Thakur, Utkal Mohanty, R. S. Dhaliwal

**Affiliations:** 1 Oral Health Sciences Centre, PGIMER, Chandigarh, India; 2 Indian Council of Medical Research, New Delhi, India; 3 Division of Neonatology, Department of Pediatrics, PGIMER, Chandigarh, India; 4 Department of Biostatistics, AIIMS, New Delhi, India; 5 Foundation of Healthcare Technologies Society, New Delhi, India; 6 Community Medicine and School of Public Health, PGIMER, Chandigarh, India; 7 Department of Community Dentistry, SCB Dental College & Hospital, Cuttack, Odisha India

**Keywords:** Caregivers, Child, Community health workers, Dental caries, Health education, Dental, Streptococcus mutans

## Abstract

**Background:**

Early childhood caries (ECC) has reached epidemic proportions affecting millions of children worldwide. Its prevention becomes imperative owing to the significant morbidity and financial implications involved with its treatment. The Integrated Child Development Scheme (ICDS), launched in India to provide mid-day meals, pre-school education and primary healthcare to children, can be utilised to counsel and deliver oral health education to mothers. The purpose of the study is to compare the effect of an oral health care package (OHCP) with usual care on the change in dental disease status among 1 to 3-year-old children at Anganwadi centres (AWC) in periurban areas of Chandigarh and rural areas of Cuttack, Orissa over a follow-up period of three years.

**Methods:**

Two geographically distant ICDS blocks would be selected at each of the two study sites and would be randomly allocated to intervention and control group. Closely located AWCs under each of the selected blocks shall constitute the study setting. OHCP would be delivered to the mothers of the 1–6-year-old children enrolled in the AWCs of the experimental group whereas mothers under control group would receive usual care advice available at the AWCs.

**Discussion:**

ECC prevention had conventionally focused upon testing effectiveness of programs targeting behaviour change among the caregivers and children, but surprisingly minimal efforts have been made to seek translation of these efforts into reduction of ECC at the community level. The present study has two components; testing effect of altering maternal and child behavioral aspects on ECC incidence through cohort follow up of 1–3-year-old children for three consecutive years and cross-sectional follow up of all available 1–6-year old children at the selected AWCs at regular intervals to look for change in prevalence of ECC at community level. In other regions of the world surveys of ECC prevalence before and after the intensive educational programs have shown a significant reduction in ECC prevalence. A similar decline can be anticipated through this program.

**Trial registration:**

This trial has been prospectively registered at Clinical Trials Registry, India (CTRI/2019/02/017556, 08 February 2019).

## Background

Early childhood caries (ECC) is a significant public health challenge that has reached epidemic proportions and has affected millions of young children both in developed and developing nations [1–3]. While considering the potential burden and the morbidity associated with this disease, several policies have been formed and implemented at the international level to decrease the overall burden of the disease across various age groups by setting age-specific targets. A joint group of the World Health Organization (WHO) and the Federation Dentaire Internationale (FDI) proposed the global average for dental caries to be 3 DMFT for 12-year-olds and 50% of 5–6-year-olds to be free of dental caries by 2000 [4]. Prevalence of dental caries is on a decline in many industrialised nations due to their well-structured oral health programs, and a good percentage of their GDP had been directed towards oral health, and the majority of them have achieved these goals. However, the developing nations like India are far from reaching WHO targets due to lack of any national-level policies for prevention and control of dental diseases. The problem is compounded by several factors including the disparity in the distribution of population, with two-thirds of the Indian community residing in rural areas, of which 40% are children [5]. The dramatic improvement in the dentist-population ratio over the past 6–7 decades from 1:301,000 in the 1960s to an average of 1:9992 in 2016 has not equally reflected in the rural and urban areas with the majority being concentrated in the latter (1:4000) and only about 25–30% of them serving in the rural regions (1:30,000); consequentially even necessary oral health facilities are not available in some of the areas [6]. The prevalence of ECC in Indian children less than 5 years ranges between 50 and 70%; the significant sequelae being root stumps warranting extractions and abscesses resulting from pulpal involvement of primary teeth, ultimately affecting the speech, mastication and esthetics of the child [7–11]. Literature regarding the impact of ECC on the quality of life of children as well as their families reports that despite comprehensive oral rehabilitation of the affected dentition, their quality of life remained significantly lower than that of children who did not develop the disease [12]. Keeping in mind the significant morbidity, loss of school hours in children and substantial finances involved in the treatment of ECC, the only practically viable solution for dealing with ECC is its prevention.

Prevention of ECC can be brought through education of expectant mothers and mothers of young children regarding appropriate oral hygiene measures and dietary practices. In a utopian universe, this task would best be carried out by trained dental professionals. However, the country faces a gross disparity in the regional distribution of the dental health workforce, as mentioned before. A promising alternative workforce for the task seems to be the female workers at the Anganwadi centres (AWC) under the ICDS scheme which was launched in 1975 to provide mid-day meal, pre-school education and primary healthcare to children under 6 years of age. These workers can be used to effectively counsel and deliver the oral health education to both children and mothers’ thus serving as the much-needed initiative at the national level. ICDS scheme is an ideal example of community participation under the Primary Health Care concept (Alma Ata declaration 1978). As of 2015, the number of operational AWCs in India is 1,349,091 with each centre covering a population of 1000 in rural and urban areas and 700 in Tribal regions [13]. Anganwadi workers (AWW) promote and monitor overall child development, carry out additional feeding, immunisation, dispense vitamin A, iron and folic acid tablets and referral to the medical care facility. AWWs also provide outreach services by visiting door-to-door in the community as agents of social change mobilisation for better care of young children, girls and women. Though adequately trained on issues concerning general health, various studies have cited limited oral health knowledge and awareness among them [14, 15]. Nevertheless, they appear to be a potential health workforce that is yet to be utilised for controlling and limiting many prevalent oral diseases like ECC in India.

A one-year pilot project funded by the Department of Science and Technology, Government of India (2014–15), was carried out to develop an oral health care package for prevention and control of oral diseases among mothers and pre-school children, and to test whether AWWs could be utilised for delivering this package.

The present research project aims to compare the effect of the above tested oral health care package versus routine dental care advice generally imparted by the AWWs on change in dental caries, gingival health and S mutans levels among 1 to 6-year-old children and KAP regarding oral health among their mothers at AWCs. The study shall be simultaneously carried out at two geographically distant sites; peri-urban areas of Chandigarh and rural parts of Cuttack, Orissa. In contrast to Chandigarh, rural Cuttack has got a significant proportion of the tribal population with differing socio-economic, cultural background and dietary habits etc. Keeping two sites in the study shall also additionally permit comparison of study outcomes between these two different populations [16, 17].

## Methods

### Study design

This will be a pragmatic, two-arm, parallel-group, community-based controlled trial with clusters of AWCs within ICDS blocks as the unit of allocation. Two geographically distant ICDS blocks will be selected at each of the study sites, viz. Chandigarh and Cuttack. The selected blocks will be randomly allocated to the intervention and control group using sealed envelopes by a statistician who will not be the part of this study. AWCs under each of the two selected blocks shall be enumerated and, from them, one AWC will be randomly selected, and further AWCs located adjacent to this AWC will then be chosen so that a cluster of AWCs is formed within the block. This cluster of AWCs will constitute a unit of allocation. A typical cluster-randomised controlled trial should have a large number of small-sized clusters, randomly allocated to an intervention and control arm. However, for logistic reasons, we have opted for a pragmatic design in which only two large-sized clusters at each study centre (Chandigarh and Cuttack) would be compared.

### Study participants, study setting and subject eligibility criteria

Children aged 0–6 years and their mothers attending AWCs shall constitute the study population. Expectant mothers and mother-child pair of children aged 0–6 years enrolled at the selected AWCs and who agree to participate in the study will constitute the study sample. Study subjects will be excluded if theyare found to be already enrolled with any other trial related to oral health care.

### Study groups and intervention

#### Group I (intervention group AWC)

Oral Health Care Package (described below) for primary prevention of oral diseases will be delivered to the study participants in this group.

#### Group II (control group AWC)

Participants will receive the standard care, i.e. the routine dental care available at the AWCs. The prevailing standard of oral health care in AWCs is part of maintaining complete hygiene of the body, wherein the AWW gives basic instructions to maintain oral hygiene too.

### Components of Oral health care package

#### Oral health education

Training sessions of 4–5-h duration will be conducted to train AWWs in batches. These sessions will focus on oral health primary preventive strategies using pre-tested standardised oral health education materials (pictorial flip charts, posters, models, pamphlets/brochure in local language) and practical demonstrations on gum pad cleaning, tooth brushing and plaque control using disclosing agents. Pre- and post-training evaluation of the AWWs will be done, and re-training sessions will be conducted for those who fail to reach a cut-off score of 90%. These trained AWWs will then provide oral health education to mothers on (a) proper feeding habits, (b) role of feeding in causation of ECC, (c) oral hygiene measures including gum pad cleaning, (d) types of toothbrushes, their role in maintaining oral hygiene, frequency of brushing per day, time to initiate tooth brushing (e) use of fluoridated toothpaste – its amount, use and frequency (f) role of parents in maintaining oral hygiene of the child, (g) intelligent use of sugars, (h) transmission of S mutans (i) abnormal oral habits, teething, bruxism, dental traumatic injuries etc.

#### Application of 2.26% fluoride varnish and 10% Povidone-iodine

2.26% fluoride varnish and 10% Povidone-iodine will be topically applied to the teeth of children aged 1–6 years, once every 3 months, by a dentist in the same appointment

#### Weekly Oral health education Mobile messages

Mothers, who will subscribe to receiving weekly SMSs, will receive message tips on oral health promotion and disease prevention. These messages will be customised based on the age of the child as well as the presence of risk factors for dental caries and other oro-dental diseases.

### Sample size estimation

Sample size calculation for the study has been based upon a similar study conducted by Lawrence HP et al., 2008 for prevention of dental caries among aboriginal children in Canada, with 2-year, follow up [18]. In the above study, the mean (SD) change of dmfs in intervention and control arm was 11 (14.3) and 13.47 (16.3), respectively, resulting in a mean difference of 2.47.

Assuming a 5% alpha error and 20% beta error, to detect a mean difference with standard deviations as above, using the sample size formula (z_1-α/2_ + z_1-β_)^2^ x (σ_1_^2^ + σ_2_^2^)/(μ_1_-μ_2_)^2^, the required sample size has been calculated to be approximately 605 in each arm. The design effect due to clustering has not been included, because we do not have prior information about the intra-class correlation within a cluster. To account for an attrition in the number of study subjects, and to account for a decrease in the effect size (due to a potential waning in the performance of AWWs in the intervention clusters over the three-year study period, and a possible Hawthorne effect) it was decided to inflate the sample size approximately 1.5 times, resulting in 950subjects in each arm at each study center (Chandigarh and Cuttack). On average, around 25 children are attached to each AWC. Therefore, at each study centre, a cluster of 40 AWCs will be selected within each of the two ICDS blocks, and the blocks will be randomly located to the intervention and the control arm.

### Plan of study (Fig. 1)

#### Measurement of variables

The following demographic variables and potential confounders will be recorded at enrollment; socio-economic status of children, number of siblings, order and age of children, child anthropometry (weight & BMI), eating patterns, oral health services utilisation by children and health behaviors of children, maternal and child medical history and health literacy levels of mothers etc.

**Fig. 1:**
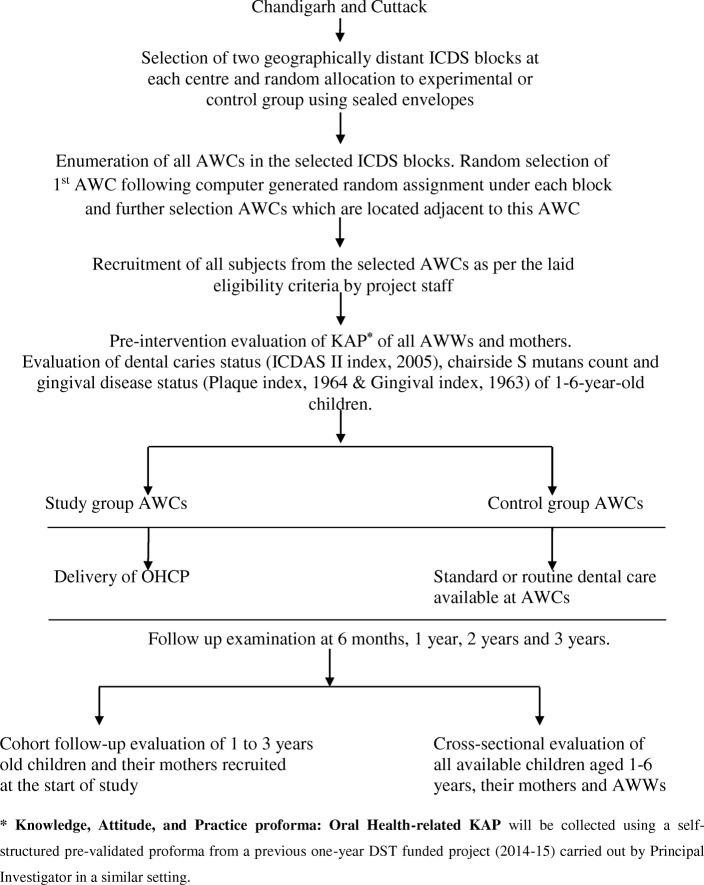
Plan of study

### Co-interventions

The following co-interventions are present in this study; supplementary nutrition, health education, pre-school non-formal education, immunisation, health check-up and referral services. These services are available to children in both the intervention and control arm as an inherent part of the ICDS scheme [13].

### Outcome variables

These will be assessed at enrollment, 6 months, 1 year, 2 years and 3 years after the start of the project. Personnel assessing the outcomes will be blinded to the group of intervention. The primary outcome variable will be change in dmfs count among 1 to 3-year-old children over a follow-up period of three years.

The secondary outcome variables will be:

- individual child-level variables: dental caries count (caries reversals and caries increment), S mutans levels, Gingival and Plaque Index
- mother and AWW-level variables: oral health-related KAP

Variables that will be measured at baseline and at each of the above time points are shown in Table 1.

**Table 1:** Variables to be assessed during the study

Variables Assessment	Variable types	Data Analysis	Anticipated Outcomes	Time of measurement
At the level of the mother (Independent variables)				
Socio-Demographics	Continuous & Categorical	T-test and Chi-square tests.	Assess the distribution of population characteristics	At baseline
Health Literacy Levels	Continuous	T-test	Assess health-related literacy of the individual	At baseline
At the level of the child (Potential Confounders)				
Child Anthropometry (Weight & BMI)	Continuous	T-test	Assess BMI	At baseline and 6 months, 1 year, 2 years and 3-years post-intervention
Oral Health services utilisation	Categorical	Chi-square test	Assess the utilisation of dental care in the last 12 months	At baseline and 6 months, 1 year, 2 years and 3-years post-intervention
At the level of Mother and Anganwadi Worker (Outcomes)				
Oral Health-related KAP	Continuous & Categorical	T-test and Chi-square test	Assess the level of KAP related to oral health issues	At baseline and 6 months, 1 year 2 years and 3-years post-intervention
At the level of Child (Outcomes)				
Dental caries count	Continuous and Categorical	T-test and Chi-square test	Assess the level of dental caries status	At baseline and 6 months, 1 year and 3-years post-intervention
S. mutans levels	Continuous and Categorical	T-test and Chi-square test	Assess the level of dental caries causing organisms	At baseline and 6 months, 1 year and 3-years post-intervention
Gingival and Plaque Index	Continuous and Categorical	T-test and Chi-square test	Assess the level of oral hygiene	At baseline and 6 months, 1 year and 3-years post-intervention

### Plan for analysis

As the population of children in an AWC is not stable, with older children who cross 6 years of age leaving the AWC and young children joining the AWC; two types of analysis have been planned. The primary analysis will be an inter-group comparison of a stable cohort of children aged 1 to 3 years who will be recruited at the beginning of the study and shall be followed up for 3 years until the age of 4 to 6 years respectively. The secondary analysis will be a cross-sectional inter-group comparison of all available children aged 1 to 6 years present at follow up evaluations (6 months, 1 year, 2 year and 3 years). Children in the secondary analysis will have varying points of entry and different durations of follow-up. All the above-listed outcome variables will be analysed both in the primary and the secondary analysis.

The primary analysis will be done on an intention-to-treat basis. Qualitative data shall be expressed using frequency distribution and percentages, whereas quantitative data shall be expressed as mean (standard deviation) or median (1st, 3rd quartile) depending upon distribution. Normality of distribution will be assessed by Shapiro Wilk test and Q-Q plot. Qualitative variables will be compared between the two groups by chi-square test or Fisher’s exact test, as appropriate. Quantitative variables will be compared between the two groups by Student’s t-test or Mann-Whitney U test, depending upon distribution. Repeated measures analysis between the two groups will be performed using two-way repeated-measures ANOVA or mixed linear models or generalised estimating equations, depending upon the variable and statistical assumptions.

### Data monitoring, quality assurance and auditing

The real-time data entry would be done using a mobile tablet-based data collection platform. To ensure project efficiency we will have (a) well trained team of dentists and field workers (b) calibration of dentists and field workers before commencement of study, at 6 months, 1 year and 2 years (c) outcome assessors who will be blinded for all follow up examinations (d) geographical distance between the ICDS blocks to avoid contamination, (e) weekly meetings with the research staff, (f) logs of all study participant contacts. To ensure efficient and accurate data management, the electronic data collection system will centrally collect all the data on the server and have an administrative account to (i) maintain records of all the data gathered from the participants during each visit (ii) central data processing and (iii) fortnightly data checks. Security of the data will be maintained through regular backups, and all computers and specific data files will be password protected and kept in a locked file cabinet. Only the project Principal Investigator will have access to this administrative account.

Further, a Data Monitoring Committee independent of the study sponsors and headed by Principal Investigator at the Technical Co-ordinating Centre shall carefully monitor the project during its course. Adverse events if any shall be carefully looked upon, documented and reported to the relevant authorities, if that warrants any treatment shall also be taken care off. Subjects who encounter any significant adverse event shall be excluded from the trial, and a noting shall be made for the same. The committee shall also carry out periodic interim assessments to review further course of study, shall look upon any deviations required from the protocol. Committee will further submit its report to the funding agency task force, which would take the final decision on deviations from the protocol and if trial needs to be terminated at any point of time. Such deviations if any, shall be communicated to all the stakeholders including investigators from all the participating sites, funding agency, institutional ethics committee, Clinical Trials Registry, India, study participants, protocol publication journal. This funding agency task force headed by an independent external expert would carry out annual project auditing as well. At the end of the project, detailed report about project outcomes shall be reviewed by the funding agency taskforce and the outcomes may be presented to policymakers at central as well as state levels. Project results shall be disseminated through scientific research publications.

### Management of oral diseases encountered during oral health examination of children

Adequate referral of participants to the nearest government hospital or dental college would be done by project staff for the management of oral diseases detected during the study. Referral cards mentioning oral examination findings and a provisional diagnosis of the child would be given to the mothers.

## Discussion

Although ECC has been recognised as a disease with widespread prevalence and implications but still a practical approach to manage it is far from reality. There is no scientific evidence for prevention of ECC based on a single method or to favour one approach over another [19, 20]. Hence, whole-population strategies that would disseminate information on ECC and its prevention based on current etiological paradigms of this disease are required. For obvious reasons, this dissemination should occur through the existing resources without putting any extra financial burden over the limited health resources present in developing countries. The Anganwadi workforce in India or similar primary health care setups can efficiently be utilised to implement preventive and promotive protocols to safeguard growth and development of young children. Oral disease, predominantly ECC, in young children can be prevented to a great extent if AWCs are sufficiently empowered, and the workers sensitised, educated & motivated and made responsible for educating and motivating mothers to provide the best oral care for their children [21].

Nonetheless, of the high prevalence of ECC and below-average to moderate and satisfactory knowledge of oral health among AWWs, oral health has not been included in the training package of the AWWs nationally [15]. The limited oral health-related knowledge of the AWWs would be kept in mind while designing the training module and the oral health promotion training material for the AWWs. It is expected that the AWWs’ motivation, manifested in their behaviour in the workplace, shall significantly affect the outcome of the present study. Therefore, motivation and reinforcement sessions have been kept every three months for the AWWs throughout the intervention phase of three years. AWWs remuneration pattern is honorarium based; hence, suitable honorarium shall also be given to them every month to maintain their interest in the program.

ECC prevention had conventionally focused upon testing effectiveness of programs targeting behaviour change among the caregivers and children, but surprisingly minimal efforts have had been made to seek translation of these efforts into the reduction of ECC at the community level. The present study shall simultaneously carry with it both the components, i.e. testing effect of altering maternal and child behavioral aspects on ECC incidence through Cohort follow up of 1–3-year-old children for three consecutive years and also a cross-sectional follow up of all available 1–6-year old children at the selected AWCs at regular intervals to look for any change in prevalence of ECC at community level. In other regions of the world surveys of ECC prevalence before and after the intensive educational programs have shown a significant reduction in ECC prevalence [22–27]. A similar decline can be anticipated through this program as well.

Many times, habits and behaviours are so deeply established that it becomes challenging and impractical to change them through health education. Hence, feasibility and effectiveness of delivering new approaches that are not based upon subject compliance or which specifically target behaviour change should also be evaluated. Results from professional tooth brushing programs or professional administration of topical antimicrobial agents and fluoride are ambiguous, and feasibility of any such application at large in community settings had not been discussed. The focus of the current program will be to share responsibility for caries prevention between both dental professionals and parents [1, 28–30]. Hence, in addition to educating mothers regarding occurrence and prevention of ECC, feasibility and effectiveness of the professional application of 2.26% fluoride varnish and 10% povidone-iodine in field setup shall also be assessed. Only a very few randomised clinical trials with smaller samples and shorter follow up duration have determined the effect of the application of these topical agents on ECC [18, 30–32]. Hence, the present study shall be a controlled trial that would comprehensively take into account the maternal factors (oral health education through trained AWWs and Behavior Change Communication material) and directly limiting the cariogenic bacterial count in children through professional application of 2.26% fluoride varnish and 10% Povidone-iodine. The study is also unique in terms of being amongst a very few multi-level trials with a human-centred approach to deliver oral health intervention indeed at primordial and primary level and to reduce oral diseases among pre-school children in both peri-urban and rural settings. A real-time data entry and sharing software shall be developed, and this will help in effective monitoring of the study as well. Upscaling of the project can be done to other AWCs of the country if the present approach is found to be effective and practical.

## Data Availability

The datasets generated during and/or analysed during the current study are available from the corresponding author on reasonable request.

## Abbreviations

AWC: Anganwadi centres
AWW: Anganwadi worker
dmfs: Decayed missing filled status
ECC: Early childhood caries
FDI: Federation Dentaire Internationale
GDP: Gross Domestic Product
ICDS: Integrated Child Development Scheme
KAP: Knowledge Attitude Practices
OHCP: Oral Health Care Package
S mutans: Streptococcus mutans
WHO: World Health Organization
